# A population-based comparison study of the mental health of patients with intentional and unintentional burns

**DOI:** 10.1186/s41038-018-0133-0

**Published:** 2018-11-06

**Authors:** Thirthar P Vetrichevvel, Sean M Randall, Fiona M Wood, Suzanne Rea, James H Boyd, Janine M Duke

**Affiliations:** 1 Burn Injury Research Unit, Faculty of Health and Medical Sciences, The University of Western Australia, M318 35 Stirling Highway, Crawley, Perth, Western Australia 6009 Australia; 2 Curtin Medical School, Curtin University, Perth, Australia; 3 Centre for Data Linkage, Curtin University, Perth, Western Australia Australia; 4 Burns Service of Western Australia, Fiona Stanley Hospital and Princess Margaret Hospital, Perth, Western Australia Australia

**Keywords:** Intentional burns, Mental health, Self-harm burns, Assault burns, Epidemiology

## Abstract

**Background:**

A number of studies report high prevalence of mental health conditions among burn patients. However there is a need to understand differences in the temporal relationship between mental health conditions and intentional and unintentional burns to hasten psychological prevention and intervention. This study aims to compare the socio-demographic profile, burn characteristics and pre- and post-burn psychiatric morbidity of burn patients by intent-of-injury.

**Methods:**

De-identified linked hospital, death and mental health (MH) case registry data of burn patients hospitalised in Western Australia between 1 January 1980 and 30 June 2012 were analysed. Crude (observed) post-burn rates of mental health admissions were generated by burn intent-of-injury. Descriptive statistics were performed to compare the characteristics of the burn patients.

**Results:**

A total of 30,997 individuals were hospitalised for a first burn; 360 (1.2%) had self-harm burns and 206 (0.7%) assault burns. Over the study period, admission rates for assault burns increased by 4.8% per year (95% confidence interval (CI) 3.1–6.5%) and self-harm burns increased 6.9% per year (95% CI 4.8–9.1%). Self-harm and assault burns occurred mainly among those aged 15 to 44 years (median age, interquartile range (IQR): self-harm 30 years, 22–40; assault 31 years, 23–38). Those with self-harm burns had a longer index hospital stay (median (IQR): self-harm 15 days (5–35) vs 4 days (1–11) assault vs 4 days (1–10) unintentional) and higher in-hospital mortality (7.2% self-harm vs 1.9% assault burns vs 0.8% unintentional). More than half (55.0%) of self-harm burns had a prior hospitalisation (5-year lookback) for a MH condition vs 10.7% of assault burns and 2.8% of unintentional burns. Crude post-burn rates of MH admissions per 100 person-years (PY) by intent-of-burn subgroups: self-harm 209 per 100 PY, assault burns 11 per 100 PY and unintentional burns 3 per 100 PY.

**Conclusions:**

Intentional burn patients experienced significantly higher pre- and post-burn mental health morbidity along with significant adverse outcome in comparison with unintentional burns. Early psychological assessment and intervention could help in improving the MH of these patients.

## Background

Intentional burns, encompassing self-inflicted and assault-related burns, are associated with complex psychosocial causation and significant effects on mental health (MH) [1, 2]. The incidence of intentional burn admissions varies worldwide, ranging between 1 and 10% in developed countries and 25–30% in developing countries [1, 3–6]. High incidence among young women (e.g. 63–79%) has been the characteristic of intentional burns in lower socio-economic countries [1, 5]. Intentional burns are associated with larger surface area burned and inhalation injury, resulting in longer hospital stay and increased morbidity and mortality [3, 4, 7, 8]. Owing to their severity, the management of intentional burns necessitates intense monitoring and treatment. Burn prevention strategies based on Haddon’s matrix for injury prevention differ significantly between the unintentional and intentional burns, and the need for an understanding of the human factors and behavioural patterns in these burns is important [5].

Reports of pre-injury mental illness in patients with intentional burns vary with 17.7% prevalence reported in a study from the United States of America (USA) [9] to as high as 75% [10, 11] in a study from the United Kingdom (UK). Australian data on psychiatric co-morbidity in self-inflicted burn patients has been found to be as high as 71% [12] and 73% [13] among admitted patients. Substance abuse has also been found to be high among self-inflicted (59%) [9] and assault (30%) [14] burn patients, with alcohol abuse being predominant [15]. Higher alcohol use among patients sustaining intentional burn has also been observed with 15% of those with assault burns having a measurable blood alcohol content in comparison with 7% for all other burn patients [9]. In spite of the high prevalence of pre-injury MH illness and substance abuse in patients with intentional burns, there is a scarcity of data especially in relation to assault burn injury patients who represent the severe end of the spectrum.

A burn, by itself, has been described as a risk factor for the development of psychopathological disorder, with depression and post-traumatic stress disorder being the most common [16–18]. Alcohol and substance abuse have also been found to be more prevalent post-burn [18]. Pre-burn depression, type and baseline psychiatric symptoms, percentage of total body surface area (%TBSA) burned, and pain and visibility of burn injury have been described as specific predispositions [16, 18–20]. Given the high prevalence of pre-burn MH disorders in intentional burn patients [12, 13], there is a heightened risk for occurrence and worsening of MH and substance abuse.

Pre-existing or new onset MH disorders complicate burn management because of poor compliance with medications, procedures and rehabilitation [15]. These factors, coupled with the inherent nature of intentional burns being larger surface area burns, are expected to have an additive effect on the complexity of burn management. Alcohol and substance abuse associated with intentional burns require additional intervention for dependence, withdrawal symptoms and drug interaction. Burn pain, feelings of isolation and loss of independence associated with hospitalised burn patients may worsen MH and pose challenges to the management of MH. Thus, examination of MH disorders in intentional burn injury inpatients is important as it represents a unique cohort with challenges to acute burn care, management of psychiatric illness and long-term morbidity.

We have recently examined the MH admissions of unintentional burn patients in comparison with an age and gender frequency-matched uninjured group which found significantly higher post-burn admission rates for psychiatric disorders after unintentional burns [21]. This study aims, firstly, to compare the socio-demographic and injury characteristics, comorbidity and psychiatric morbidity of people with intentional burns (both self-harm and assault) with those with unintentional burns in Western Australia, during the period of 1980 to 2012; secondly, to compare the characteristics of those hospitalised with self-inflicted burns to those with assault burns; and, thirdly, to compare the differences in post-burn admissions for MH conditions.

## Methods

This retrospective cohort study represents a secondary data analysis of de-identified linked health administrative data of burn patients hospitalised in Western Australia during the period of 1 January 1980 to 30 June 2012. Ethics approvals were granted by the University of Western Australia and the Department of Health, Western Australia. The study forms part of the Western Australia Population-based Burn Injury Project (WAPBIP) and included linked records from the Western Australian Hospital Morbidity Data System (hospital records), death register and Mental Health Information System (inpatient MH records). The Mental Health Information System is a comprehensive psychiatric case register that comprises all contacts with in-patient MH services (private and public hospitals) in Western Australia since 1966. Records were linked and extracted by the staff of the Department of Health, Western Australia Data Linkage System, an established record linkage system that routinely links whole-of-population administrative health data for Western Australia [22]. A number of papers have been published using data from the WAPBIP, and sections of the methods have been previously published [23–25].

For this study, the burn cohort comprised all persons hospitalised with an index (first) admission in Western Australia for a burn for the period 1 January 1980 to 30 June 2012. The index burn was defined using the International Classification of Diseases (ICD) codes (ICD10-AM T20-T31; ICD9-CM 940-949). Burns were classified as either self-harm (containing a code from ICD10-AM X76-77; ICD9-CM E958.1-2), assault (containing a code from ICD10-AM X97-98; ICD9-CM E968.0, E968.3) or unintentional burns otherwise.

Hospital morbidity files, MH admissions and death data were linked to each member of the burn cohort for the period 1980–2012. Indices of geographic remoteness [26] and socio-economic disadvantage [27] were also linked. The socio-economic disadvantage used in this study indicator is based on responses to 40 items in the Australian census and has shown high correlation in Australian studies with lifestyle risk factors (e.g. nutrition, physical activity, alcohol, smoking and substance abuse) [28–31]. Hospital admissions data included principal and additional diagnoses, age and gender, indigenous status, admission and discharge dates, burn characteristics (%TBSA, burn depth, site) and residential postcode.

Principal diagnosis data in the Mental Health Information System were used with ICD chapter 6 codes to identify admissions for any MH condition (ICD10 F20-F51) and drug and alcohol conditions (ICD10 F10-19), while principal external cause codes were used to identify admissions for self-harm (ICD10 X60-X84). Further analysis occurred on MH sub-conditions, including psychotic disorders (ICD10 F20-29), mood disorders (ICD10 F30-39) and anxiety-related disorders (F40-49). Equivalent ICD9-CM codes for these categories were mapped to ICD10-AM codes. Mortality data included the date and cause of death.

Charlson Comorbidity Index (CCI) [32] was generated using hospital data with a 5-year lookback period [33] (CCI = 0; ≥ 1). Variables were created to identify the number of prior admissions during the 5-year lookback period for the MH and related conditions listed above. Indices of socio-economic disadvantage (most to least disadvantaged) and geographic remoteness (major cities, inner regional, outer regional, remote and very remote) were categorised, respectively.

Length of hospital stay (LOS) for the index burn admission was generated. For post-burn analyses, individuals were followed up after index burn hospital discharge, until death or the end of the study period (June 2012). The total number of years a person was at risk (person-years) was estimated from the final discharge date. The total number of annual admissions for MH conditions prior to index and after burn discharge was also generated. The admission of the index burn was not included in these outcomes. Crude yearly admission rates were calculated from these variables for a 20-year follow-up period after index burn admission.

Descriptive statistics were generated including percentages for categorical variables and medians and interquartile range (IQR) for continuous variables. Bivariate analyses using chi-square tests for categorical variables and Kruskal-Wallis tests for continuous variables were performed to compare the patients hospitalised with self-harm burns, assault burns and unintentional burns; the level of significance was set at 0.05. Trends in yearly changes in burn hospitalisation incidence by the intent of injury were assessed using a log-linear model. All statistical analyses were performed using Stata statistical software V12 (StataCorp LP, College Station, TX, USA).

## Results

From January 1980 to June 2012, there were 30,997 individuals with a first burn hospitalisation; of these, 360 (1.2%) were intentionally self-inflicted burns and 206 (0.7%) were burns from an assault. The median length of follow-up for those with unintentional burns was 15.8 years (IQR 7.3–24.4 years, min 0 day, max 32.5 years); this compared with 7.7 years for those with self-harm burns (IQR 3.8–13.2 years, min 0 day, max 31.6 years) and 9.0 years for those with assault burns (IQR 4.7–15.2 years, min 0 day, max 32.3 years).

In 1980, the unadjusted rate of unintentional burns was 89.3 burn hospitalisations per 100,000 people, which had fallen to 43.1 burns hospitalisations per 100,000 people by 2011 (annual percentage change (APC) − 2.6%, 95% confidence interval (CI) − 3.0 to − 2.3%). However, rates of intentional burns rose over the same time period. The rate of assault burns increased by 4.8% per year over the study period (95% CI 3.1 to 6.5%), from 0.1 burn hospitalisations per 100,000 individuals in 1980 to 0.6 burn hospitalisations per 100,000 individuals in 2012. The rate of self-harm burns increased 6.9% per year over the study period (95% CI 4.8 to 9.1%), rising from 0.2 burn hospitalisations per 100,000 to 0.8 burn hospitalisations by 2011.

Table 1 compares patient socio-demographics for individuals with assault, self-harm and unintentional burns. While unintentional burns were more likely male (69%) than female, intentional burns were approximately split between genders, while individuals with self-harm burns were slightly more likely to be female (56%). Self-harm and assault burns occurred mainly in those aged 15–44 years; unintentional burns had a different age distribution, with over a third of burns occurring in those younger than 15 years (median age 23 years, IQR 7–39). Those with assault burns were more likely to be Aboriginal Australians, socio-economically disadvantaged and from very remote areas, as compared to those with self-harm or unintentional burns. Individuals with self-harm burns were generally similar to those with unintentional burns in terms of the proportion of Aboriginal Australians, but had more individuals from higher socio-economic groups, and were more likely to be from major cities. Individuals with an intentional burn (self-harm and assault) had a higher rate of previous comorbidities, which may reflect the differing age structures of the three categories.

**Table 1:** Baseline demographic factors for those with self-harm, assault and unintentional burns, Western Australia, 1980–2012

Characteristics	Self-harm burns (*N* = 360), *n* (%)	Assault burns (*N* = 206), *n* (%)	Unintentional burns (*N* = 30,431), *n* (%)	*p* value
Gender				
Male	160 (44.4)	106 (51.5)	20,957 (68.9)	< 0.001
Female	200 (55.6)	100 (48.5)	9468 (31.1)	
Age (years)				
0–14	7 (1.9)	15 (7.3)	10,414 (34.2)	< 0.001
15–24	115 (31.9)	45 (21.8)	5932 (19.5)	
25–34	106 (29.4)	67 (32.5)	4752 (15.6)	
35–44	71 (19.7)	54 (26.2)	3413 (11.2)	
45–54	36 (10.0)	18 (8.7)	2230 (7.3)	
55–64	14 (3.9)	5 (2.4)	1448 (4.8)	
65+	11 (3.1)	2 (1.0)	2242 (7.4)	
Aboriginality				
Aboriginal	40 (11.1)	108 (52.4)	4333 (14.2)	< 0.001
Non-Aboriginal	320 (88.9)	98 (47.6)	26,098 (85.8)	
Social disadvantage quintiles*				
Quintile 1 (most disadvantaged)	86 (24.1)	81 (40.5)	6412 (21.4)	< 0.001
Quintile 2	84 (23.5)	47 (23.5)	9747 (32.6)	
Quintile 3	66 (18.5)	29 (14.5)	6259 (20.9)	
Quintile 4	55 (15.4)	19 (9.5)	3759 (12.6)	
Quintile 5 (least disadvantaged)	66 (18.5)	24 (12.0)	3767 (12.6)	
Remoteness**				
Major city	266 (74.5)	91 (44.8)	15,453 (51.5)	< 0.001
Inner regional	36 (10.1)	11 (5.4)	3313 (11.0)	
Outer regional	33 (9.2)	26 (12.8)	4899 (16.3)	
Remote	9 (2.5)	14 (6.9)	3411 (11.4)	
Very remote	13 (3.6)	61 (30.1)	2937 (9.8)	
Health status				
Any co-morbidity (CCI ≥ 1)^†^	59 (16.4)	31 (15.1)	3041 (10.0)	< 0.001

*Socio-Economic Index for Areas (SEIFA) socio-economic disadvantage quintiles; missing values 1.0% burn, 0.5% no injury

**Accessibility Remoteness Index of Australia, revised version (ARIA+) remoteness classification; missing values 0.6% burn, 0.8% no injury

^†^Co-morbidity based on derived Charlson Comorbidity Index (CCI) using 5-year lookback

Individuals with self-harm burns spent longer in hospital (index admission) for the index burn (LOS median (IQR) 15 days (5–35) vs 4 days (1–11) assault burns vs 4 days (1–10) unintentional burns) and had a higher in-hospital mortality rate (7.2% (*n* = 26) self-harm burns vs 1.9% (*n* = 4) assault burns vs 0.8% (*n* = 253) unintentional burns). The vast majority of cases were discharged home across all burn intention categories. Those with assault burns were more likely to leave against medical advice (7.5% (*n* = 15), compared with 2.3% (*n* = 8) for self-harm and 0.9% (*n* = 257) for unintentional burns), while those with self-harm burns were more likely to be discharged to psychiatric care (7.6% (*n* = 27) compared with 2.5% (*n* = 5) for assault burns and 0.4% (*n* = 132) for unintentional burns).

Comparisons of burn characteristics are presented in Table 2. The proportions of full thickness burns and those with burns of ≥ 20% TBSA were higher among self-harmers as compared to assault and unintentional burns. Among self-harm burn patients, males (69.5%) were more likely than females (30.5%) to have more extensive burns of TBSA ≥ 20% (refer to Table 3). For 49.1% (*n* = 15,232) of the cohort, TBSA was ICD coded ‘unspecified’, and typically, these individuals had a burn admission earlier in the study period (~ 1980–1996, ICD9 codes). However, median LOS (IQR) for cases with unspecified TBSA of 3 days (1-10) was found to be similar to that for cases with burns < 20% TBSA of 4 days (1-10), suggesting unspecified TBSA cases were most likely less severe (< 20%TBSA). Individuals with severe burns (≥ 20%TBSA) generally had longer median LOS of 25 days (12–48).

**Table 2:** Burn characteristics for those with self-harm, assault and unintentional burns

Characteristics	Self-harm burns (*N* = 360), *n* (%)	Assault burns (*N* = 206), *n* (%)	Unintentional burns (*N* = 30,431), *n* (%)
Burn depth*			
Erythema	53 (14.7)	36 (17.5)	3250 (10.7)
Partial thickness	122 (33.9)	92 (44.7)	10,957 (36.0)
Full thickness	106 (29.4)	38 (18.4)	4246 (14.0)
%TBSA *			
0–9%	253 (70.3)	128 (62.1)	13,214 (43.4)
10–19%	12 (3.3)	16 (7.8)	1226 (4.0)
≥ 20%	59 (16.4)	23 (11.2)	829 (2.7)
Burn location			
Head/neck	56 (15.6)	70 (34.0)	6258 (20.6)
Torso	86 (23.9)	110 (53.4)	7066 (23.2)
Arms/hands	284 (78.9)	101 (49.0)	12,759 (41.9)
Legs/feet	88 (24.4)	56 (27.2)	10,393 (34.2)
Other	13 (3.6)	8 (3.9)	2867 (9.4)
Burn cause			
Fire/flame	301 (83.6)	102 (49.5)	10,734 (35.3)
Scald	59 (16.4)	104 (50.5)	11,228 (36.9)
Other/unknown	0	0	8469 (27.8)

*Missing values relate to burn records with International Classification of Diseases (ICD) code ‘unspecified’ depth and total body surface area (TBSA)

**Table 3:** Gender breakdown of total body surface area (TBSA) classification for self-harm burns

%TBSA	Male (*N* = 144), *n* (%)	Female (*N* = 180), *n* (%)	Total (*N* = 324), *n* (%)
< 10	96 (37.9)	157 (62.1)	253 (78.1)
10–19	7 (58.3)	5 (41.7)	12 (3.7)
≥ 20	41 (69.5)	18 (30.5)	59 (18.2)

Self-harm burns occurred largely on the arms and hands (78.9% of cases) as compared to other burn types; assault burns were more likely to occur on the torso than other burn types. Intentional burns were exclusively either caused by fire or scalds; self-harm burns were caused by fire in over 80% of cases, while assault burns were split evenly between flame and scalds. For unintentional burns, 27.8% of cases resulted from other causes, most predominantly contact burns (10.6%).

As shown in Table 4, over half (55.0%) of those with self-harm burns had a hospitalisation in the previous 5 years for a MH condition (primary diagnosis), compared with 10.7% of individuals with assault burns and 2.8% of those with unintentional burns. Over a third of those with self-harm burns had a previous hospitalisation for self-harm, a proportion much higher than that found in those with assault or unintentional burns. The proportion of individuals with previous admissions for conditions related to drugs and alcohol was higher for those with intentional burns compared to those with unintentional burns. Those with self-harm burns with previous hospitalisations for MH and related conditions typically had a higher number of hospitalisations within the 5-year window as compared to individuals with assault/unintentional burns.

**Table 4:** Mental health (MH) status prior to burn injury for those with self-harm, assault and unintentional burns

Characteristics	Self-harm burns, *N* = 360	Assault burns, *N* = 206	Unintentional burns, *N* = 30,431	*p* value
Number of individuals (%) with hospitalisation in 5 years prior to burn injury for:				
Any MH condition	198 (55.0)	22 (10.7)	847 (2.8)	< 0.001
Psychotic disorder	50 (13.9)	3 (1.5)	238 (0.8)	< 0.001
Depressive condition	129 (35.8)	16 (7.8)	422 (1.4)	< 0.001
Anxiety condition	109 (30.3)	10 (4.9)	403 (1.3)	< 0001
A self-harm admission	126 (35.0)	13 (6.3)	503 (1.7)	< 0.001
A drug/alcohol admission	58 (16.1)	30 (14.6)	618 (2.0)	< 0.001
Any of the above	224 (62.2)	45 (21.8)	1477 (4.9)	< 0.001
Median number of hospitalisations (interquartile range, IQR) for:				
MH admissions for those with at least one MH admission	3 (1–8)	2 (1–4)	2 (1–3)	< 0.001
Self-harm admissions for those with at least one self-harm admission	3 (1–6)	1 (1–2)	1 (1–2)	< 0.001
Drugs/alcohol admissions for those with at least one drug/alcohol admission	2 (1–3)	1 (1–2)	1 (1–3)	0.030

**Fig. 1:**
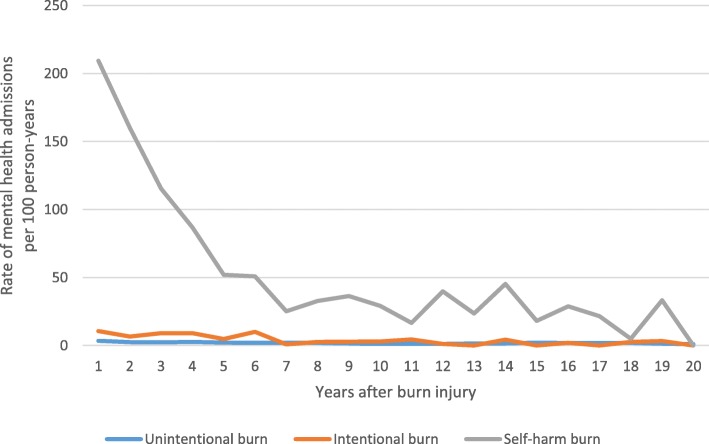
Observed (unadjusted) rates of mental health hospitalisations per 100 person-years for those with self-harm, assault and unintentional burns, Western Australia, 1980–2012

The crude (observed) rate of MH admissions for the 20 years after burn injury, stratified by burn intention, is shown in Fig. 1. Individuals with self-harm burns had a greater proportion of MH admissions after burn injury as compared to assault and unintentional burns; in the year after burn injury, those with a self-harm burn had 209 MH hospital admissions per 100 person-years (PY), while those with assault and unintentional burns had 11 and 3 MH hospitalisations per 100 PY, respectively.

Crude admission rates for self-harm, mental and behavioural conditions related to drugs and alcohol and MH sub-conditions for the 5 years after burn injury are shown in Table 5. Those with self-harm burns typically had the highest admission rates for all sub-conditions as compared to the other burn categories. Individuals with assault burns had higher admission rates than those with unintentional burns for mood and anxiety disorders, mental and behavioural disorders due to drugs and alcohol, and self-harm, with rates of drug/alcohol-related disorders closest to the rates found in those with self-harm burns. In general, these rates mimic the patterns found in hospitalisations prior to the burn injury (see Table 4). Five-year mortality rates for those who survived the initial burn admission were highest for those with assault burns (6.4%; 13 deaths out of 202 remaining individuals), followed by self-harm burns (5.7%; 19 out of 334 individuals) and unintentional burns (4.9%; 1489 out of 30,178 individuals). Unintentional injury was the most common cause of death for those with assault burns, accounting for 5 of the 13 deaths; for those with self-harm burns, the most common cause of death was suicide (5 of the 19 deaths) followed by unintentional injury (another 4 of the 19 deaths). For those with unintentional burns who died within 5 years after burn injury, suicide accounted for 50 deaths, while unintentional injury accounted for 108 deaths.

**Table 5:** Crude (observed) rates of hospitalisations for specified conditions per 100 person-years in the first 5 years after burn injury

	Year after burn injury				
	Year 1	Year 2	Year 3	Year 4	Year 5
Mood disorders (rate per 100 person-years)					
Self-harm burn	133.1	86.1	68.7	37.4	21.8
Assault burn	6.6	3.8	7.9	5.8	1.4
Unintentional burn	1.2	0.9	0.8	0.9	0.8
Anxiety related disorders (rate per 100 person-years)					
Self-harm burn	57.2	61.4	33.4	39.0	17.5
Assault burn	3.0	1.1	0.6	3.2	2.7
Unintentional burn	1.3	0.8	0.8	1.2	0.6
Psychotic disorders (rate per 100 person-years)					
Self-harm burn	18.1	10.2	12.1	10.0	12.8
Assault burn	1.0	1.6	0.6	0.0	0.7
Unintentional burn	0.9	0.7	0.7	0.6	0.7
Mental and behavioural disorders due to drugs/alcohol (rate per 100 person-years)					
Self-harm burn	17.1	8.5	7.8	7.7	4.3
Assault burn	3.0	8.8	6.1	3.2	8.1
Unintentional burn	1.9	1.4	1.3	1.0	0.9
Self-harm (rate per 100 person-years)					
Self-harm burn	62.4	42.0	31.7	32.0	28.2
Assault burn	3.0	2.7	1.8	1.9	3.4
Unintentional burn	0.9	0.8	0.6	0.4	0.5

## Discussion

While intentional burns represent a small proportion of all burn admissions in Western Australia, annual admission rates increased by 4.6% per year for assault burns and 6.9% per year for self-harm burns from 1980 to 2012. This is a concerning trend as admissions for unintentional burns have declined at a rate of 2.6% per year over the same time period. Our cohort of intentional burns had a higher median age (self-harm 30 years; assault burns 31 years) in comparison to the unintentional burn (23 years), findings similar to a previous Australian study (average age for self-harm—30 years) [34]. However, this age was lower than that reported in other studies. In the UK, a study reported a mean age of admitted patients for self-inflicted burns of 37 years and for assault burns, 29 years [35]. Worldwide, the average age at the time of injury reported for intentional burns ranges from 23 to 49.5 years [4, 10, 36–39]. Unintentional burns were more frequent in males when compared with intentional burns, with self-harm burns more common in females (56%). The male preponderance among self-inflicted burn patients has been reported in studies from UK [10, 35], Australia [34] and USA [6]; however, the gender ratio varies [38].

Those with self-harm burns were more likely to have full thickness burn sites recorded with a large proportion of burns occurring on the arms and hands. A greater proportion of those with self-harm burns (16%) had severe burns (≥ 20% TBSA) when compared with assault and unintentional burns. The majority of self-harm burns were < 10% TBSA and may reflect cases of those who had deliberately hurt themselves without suicide attempt; females represented 62% of this TBSA classification. Males represented 70% of self-harmers with severe burns ≥ 20% TBSA, a finding consistent with another Australian study of self-inflicted burns where suicide attempters were more likely to be male, with self-immolation with flammable liquid resulting in major burns and high in-hospital mortality [34].

This increased severity of burns is presumed to be an important cause of longer hospital stays (index admission) and mortality observed among self-harm burns [40]. In this study, LOS was markedly different between self-harm burns (median 15 days), assault burns (4 days) and unintentional burns (4 days), similar to the patterns reported in another Australian study (average LOS self-inflicted burns of 52 days vs 12 days for the burn unit average) [13]. In-hospital mortality among those with self-harm burns and assault burns in this study was nine times and four times higher, respectively, than that identified for those with unintentional burns (0.8%). The longer LOS observed for self-harm burn patients most likely reflects the severity of burn depth and surface area burned as well as a number of other factors including mental health management and social/environmental factors including ascertaining appropriate discharge arrangements to home or another suitable medical facility for recovery. Approximately 30% of the self-harm cohort had full thickness burns which would have required surgical intervention. In addition, this patient subgroup would also need prolonged psychiatric management and in-hospital stay to ensure the stability of the patient before discharge.

Presence of MH issues is considered a significant predisposing factor in self-harm burns. There were significantly higher counts of hospital admissions for MH issues among self-harm (55%) patients when compared to assault (10.7%) and unintentional burn (2.8%) patients. Similarly, high reports of MH prevalence (55%, 65%) have been reported among self-harm burn patients in studies from the UK [10, 35]. The prevalence of psychiatric disease among assault patients was also similar to that found in other studies in the UK (12.2%) [35] and in Brazil (50% in self-inflicted, 10% in assault, 3.4% in unintentional) [40]. High incidence of MH issues (73%) has been reported among self-injury burn patients in comparison with all (both intentional and unintentional burns (4%)) in a study from New South Wales, Australia [13]. In Australia, the majority of burn admissions are for unintentional burns [41, 42]. Nevertheless, the prevalence of pre-existing psychiatric disorders among intentional burn patients is high, although estimates are variable [15, 35, 43]. MH medical care visits during the 12-week period prior to the injury admission have also been reported to be associated with an intentional burn admission [44].

Depressive conditions were the most commonly identified pre-burn mental illness, followed by anxiety and psychotic disorders. However, the occurrence of each of these types of mental illness was highest among the self-harm group followed by those with assault and unintentional burns, with the trend being statistically significant. This is a general trend reported in numerous studies [13, 40, 45]. Davidson et al. found the incidence of depression in self-harm patients (31%) to be similar to our results (35.8%); however, psychotic illness (19%) [10] was marginally higher than that in this self-harm burns cohort (13.9%). Among assault victims, the occurrence of depression (7.8%) was found to be similar to that reported in studies from the UK (9.8%); however, the incidence of psychotic disorder (schizophrenia) reported was higher (2.4%) than that found in this study (1.5%) [35]. Among self-immolation patients in low-income countries, adjustment disorder is the most common psychiatric disease, while in more economically developed countries, prevalence of major mental illness (18–92%) is significant [2, 6].

Pre-existing alcohol/drug abuse was significantly higher among self-burns (16.1%) and assault burns (14.6%) patients in comparison with the unintentional burns (2%). Most other studies from the UK and USA have reported higher alcohol/drug use (25 to 36%) [14, 15, 35, 40], while lower prevalence has also been reported [10]. A prior history of self-harm admission was significantly higher (35%) in our self-harm burn cohort in comparison to the assault (6.3%) and unintentional burn (1.7%) cohorts, results consistent with other studies [15, 40].

The observed (unadjusted) admissions rates for the self-harm group during the first year post-burn were substantially higher for mood- and anxiety-related disorders than that for psychotic and drug/alcohol-related disorders. Among those with assault burns, mood disorder was the most common MH condition followed by anxiety/drug-alcohol related and psychotic disorders. The observed rates of admissions for MH disorders for those with unintentional burn patients were lower than that for intentional burn patients. However, to keep these results in context, our previous research examining unintentional burns identified adjusted admission rates for MH conditions to be almost five times higher than that of an age and gender-matched uninjured cohort (IRR 4.89, 95% CI 3.52–6.69), for the first 5 years post-burn [21]. Overall, the observed admission rates for all MH sub-conditions were substantially higher among self-harm burn patients in relation to patients with assault and unintentional burns.

Palmu et al. [16] reported that, at the end of 6 months follow-up after burns, the prevalence of mood disorder was the highest (32.6%) MH issue followed by alcohol/substance-related disorder (15.2%), anxiety disorders (14.1%) and psychotic disorders (5.4%). A relatively high prevalence of substance abuse disorders has been found among burn patients (unintentional and intentional) [16, 46], consistent with the results found in this study and in our previous post-unintentional burns MH study [21].

Recently, two Canadian studies using linked health data identified a high prevalence of psychiatric morbidity among burn patients; however, no significant change in MH care utilization before and after burn injury was found [44, 46]. The differences in study findings are most likely related to the study design used and the type of health data collected. However, Mason et al. observed that those with one or fewer MH visits before burn admission had a significant (threefold) increase in post-burn MH visits, along with similar significant increases in visits related to substance abuse, mood disorders, schizophrenia, self-harm and anxiety post-burn injury [44].

The worsening of pre-existing psychiatric morbidity in intentional burns has been attributed to several causes including anxiety to acute pain, alcohol/drug withdrawal and need to administer strong analgesics with sedative effect [6, 18]. Evolving research shows that peripheral inflammation, cell-mediated immune activation and oxidative stress have been found to contribute to depressive symptoms and anxiety-like behaviour [47]. In addition, meta-analyses have also provided evidence that other major psychiatric disorders are often accompanied by activation of inflammatory and cell-mediated immune pathways, for example, mania and bipolar disorder [48] and schizophrenia [49]. Burns have been shown to elicit local and systemic inflammation and immune changes along with prolonged periods of oxidative stress which may exacerbate pre-existing MH disorders and/or lead to new symptoms or disorders [50, 51].

While it is highly likely that people with pre-existing mental health issues, prior admissions for MH and or self-harm injury, will have contact with mental health experts, these results have implications for health policy and clinical management of burns during the index admission, out-patient follow-up and longer-term MH assessment of burn patients via primary care. It is possible that self-report patient health surveys during index admission and after discharge that includes questions about psychological health may be beneficial in identifying MH care needs of patients. However, self-reported follow-up data may be sporadic and incomplete. Likewise, for such information to be effective, burn patients need to be connected to psychological/psychiatric support within the health service team, to respond to such MH patient needs. After healing of burn wounds and ending of surgical follow-up, it may also be beneficial for patients to have regular health assessments with their primary care physician that may subsequently result in better management of MH needs and also reduce the need for hospital admissions.

### Study strengths and limitations

Our ‘whole-of-population’ study measured pre- and post-burn psychiatric morbidity in intentional and unintentional burn patients based on hospitalisations for MH conditions with long follow-up. The population-based health administrative databases do not routinely contain clinical details for each individual including mental health interventions (therapies or medications) and functional status or return to work. Such data are more likely to be stored in specific clinical in-hospital and post-discharge follow-up databases, collected by clinical staff and/or self-reported health surveys. Our findings may under-represent the true magnitude of MH disease among both intentional and unintentional burn patients, as primary healthcare data and medication use, was not included in the study. In addition, using hospital admissions for MH conditions as the measure of psychiatric morbidity, the study findings relate to more severe cases. For the earlier period of the study (approximately 1980–1996), there was a high proportion of burn records without a supplementary ICD TBSA code. However, the majority of these unspecified TBSA burns were in the unintentional burn subgroup, and comparative studies of LOS of the index admission suggested that the majority of unspecified TBSA burns were most likely less severe (< 20%TBSA). The use of linked population-based health administrative data from a number of sources provides a useful platform to examine and quantify the longer-term health of all burn patients in a timely and cost-efficient way, results of which can subsequently support changes in clinical management and health policy. Future pathways that expedite linkage of statewide databases with national pharmaceutical claims and primary care databases will help to provide more complete data on the health experience of burn patients after discharge.

## Conclusions

Although patients with intentional burns represent a small proportion of all hospitalised burn patients in Western Australia, this population-based study has shown that there is a high pre- and post-burn prevalence of MH disorders among intentional burn patients, especially among patients with self-harm burns in comparison with those with unintentional burns. The burn characteristics and high prevalence of psychiatric co-morbidity associated with intentional burns resulted in adverse acute in-hospital and long-term health outcomes. This emphasises the need for adequate prevention strategies, early MH evaluation and intervention for this susceptible group among the burn patients. Cognisance of the aetiology and the progression of MH issues in distinctive subsets of burn patients will hasten psychological prevention and intervention.

## Abbreviations

95% CI: 95% confidence interval
ARIA+: Accessibility Remoteness Index of Australia
CCI: Charlson Comorbidity Index
DOHWA: Department of Health, Western Australia
ICD: International Classification for Diseases
IQR: Interquartile range
LOS: Length of stay
MH: Mental health
PY: Person-years
SEIFA: Socio-Economic Index for Areas
TBSA: Total body surface area
WAPBIP: Western Australian Population-based Burn Injury Project
